# Manganese Deficiency and Mn_2_O_3_ Nanoparticles Supplementation Disrupt Bone Remodeling and Mineral Matrix Maturation in Rats

**DOI:** 10.3390/ijms27010153

**Published:** 2025-12-23

**Authors:** Ewelina Cholewińska, Jerzy Juśkiewicz, Bartosz Fotschki, Katarzyna Ognik

**Affiliations:** 1 Department of Biochemistry and Toxicology, Faculty of Animal Sciences and Bioeconomy, University of Life Sciences in Lublin, Akademicka 13, 20-950 Lublin, Poland; katarzyna.ognik@up.edu.pl; 2 InLife Institute of Animal Reproduction and Food Research, Polish Academy of Sciences, Trylińskiego 18, 10-683 Olsztyn, Poland; j.juskiewicz@pan.olsztyn.pl (J.J.); b.fotschki@pan.olsztyn.pl (B.F.)

**Keywords:** manganese carbonate, manganese (III) oxide nanoparticles, bone metabolism, bone turnover, bone-related biomarkers, gene expression, femur, rat

## Abstract

This study aimed to investigate the effects of dietary manganese deficiency and compare the impact of manganese macroparticles (MnCO_3_) and nanoparticles (Mn_2_O_3_NPs) on bone remodeling and metabolism. Twenty-seven male Wistar rats were divided into three groups (n = 9): control (standard MnCO_3_, 65 mg Mn/kg), manganese-deficient, and Mn_2_O_3_NPs-supplemented (65 mg Mn/kg). After a 12-week feeding period, bone-related markers and gene expression were analyzed in the femur and blood. Mn-deficient rats showed reduced plasma levels of bone-specific alkaline phosphatase (BALP), tartrate-resistant acid phosphatase 5b (TRAP5b), interferon-β (IFN-β), RANKL glycoprotein, 1,25-dihydroxyvitamin D_3_ (1,25-(OH)_2_D_3_), vitamin K_2_, and collagen turnover markers (PINP, CTX-1, NTX). Femur levels of BALP, TRAP5b, interferon-γ (IFN-γ), osteonectin, calcitonin, PICP, PINP, and CTX-1 were also decreased. Replacing MnCO_3_ with Mn_2_O_3_NPs increased IFN-γ but lowered IFN-β and 1,25-(OH)_2_D_3_ levels in plasma. This treatment also decreased the femur level of BALP and calcitonin, and the RANKL:OPG ratio, while increasing the expression level of *Sp7* and *Ctsk* genes. To conclude, our results suggest that manganese deficiency is associated with suppressed bone turnover and altered mineral metabolism. Furthermore, replacing MnCO_3_ with Mn_2_O_3_ nanoparticles did not yield the anticipated benefits for bone remodeling, as evidenced by the observed imbalances in osteogenic and resorptive markers, indicating a need for cautious evaluation of nanoparticle-based supplementation.

## 1. Introduction

Manganese (Mn) is an essential trace element required for maintaining skeletal health [1,2]. Its multifaceted role in bone biology primarily involves the modulation of osteoblast and osteoclast activity, regulation of enzymatic pathways responsible for bone formation and resorption, as well as coordination of bone tissue mineralization and remodeling [1,2]. Li et al. [3] demonstrated that Mn activates integrins—adhesion proteins that stimulate osteoblast proliferation, adhesion, and survival—thereby supporting the maintenance of bone mass. Moreover, Mn positively influences the expression of osteogenic genes and promotes the production of collagen and other extracellular matrix proteins [2,3]. It also plays a key role in regulating the synthesis of glycosaminoglycans, which are crucial for the structural integrity and biomechanical properties of bone [1]. In addition, Mn is essential for the proper activation of signaling pathways involved in mineralization. Moreover, it is a critical component of manganese-dependent superoxide dismutase (MnSOD), which not only protects bone cells from oxidative damage but also actively participates in the differentiation and function of osteoclasts [1].

The importance of manganese in bone tissue homeostasis is underscored by numerous literature reports indicating that a deficiency of this trace element results in impaired bone development, reduced mineral density, and increased susceptibility to deformities and fractures [1,2,4,5,6]. Strause et al. [5] demonstrated that long-term Mn deficiency disrupts calcium homeostasis and enhances bone resorption, thereby compromising mineralization. Wang et al. [7] further reported that Mn deficiency leads to architectural disturbances in the growth plate, characterized by irregular chondrocyte distribution and impaired proliferation and differentiation. Moreover, Mn deficiency may impair the expression of collagen and non-collagenous bone matrix proteins, such as osteopontin [2].

Maintaining balanced bone metabolism and remodeling, which are essential for healthy bones, requires an adequate intake of manganese (Mn) in the diet. In healthy individuals and animals consuming a diverse diet, Mn deficiency is rarely diagnosed [8]. However, it is more frequently observed in laboratory animals fed monotonous, homogeneous diets and in patients suffering from malabsorption or impaired Mn metabolism [9]. In such cases, Mn is most commonly supplemented in the form of inorganic salts, such as manganese carbonate (MnCO_3_). Although widely used, these salts exhibit low bioavailability [10].

The rapid development of nanotechnology in recent years has opened up new avenues for enhancing the bioavailability of trace elements, including manganese (Mn). Haque et al. [11] report that manganese-based nanoparticles are gaining increasing attention in biomedicine. They have been used as antimicrobial and antiangiogenic agents, antioxidants, drug delivery vehicles, fluorescence quenchers, and contrast agents for biological and MRI imaging [11]. Due to their unique physicochemical properties, biocompatibility, and ability to modulate cellular and molecular processes involved in bone repair, manganese nanoparticles have also emerged as promising tools for bone tissue engineering and regenerative medicine [12,13]. Therefore, it is plausible that they may outperform standard Mn supplements in terms of efficacy. Nevertheless, manganese-based nanoparticles raise certain concerns, as the available data regarding their effects on human and animal physiology, particularly in the context of bone health, remain inconsistent and inconclusive. Li et al. [12] indicate that Mn-doped silicate-hydroxyapatite nanowires positively affect bone tissue regeneration. These particles modulate adaptive immunity, particularly by enhancing the recruitment of CD4^+^ T cells and promoting the polarization of Th2 cells. This immunomodulatory effect is mediated by manganese superoxide dismutase (MnSOD) and the AMPK phosphorylation signaling pathway, creating an anti-inflammatory microenvironment conducive to bone healing [12]. In contrast, Westhauser et al. [13] demonstrated that mesoporous bioactive glass nanoparticles containing 5 mol% manganese (5Mn-MBGNs) support the viability and osteogenic differentiation of human bone marrow-derived mesenchymal stromal cells (BMSCs), but only at low concentrations. Importantly, higher concentrations of these nanoparticles exhibited the opposite effect, significantly impairing the viability and proliferation of BMSCs [13]. Most critically, comprehensive in vivo studies assessing the impact of dietary Mn nanoparticle supplementation on bone metabolism and turnover are still lacking. Thus, further investigations are urgently needed to elucidate the molecular mechanisms by which manganese nanoparticles affect bone tissue.

The primary objective of this study, conducted in rats, was to compare the effects of two forms of manganese—macroparticles (MnCO_3_) and nanoparticles (Mn_2_O_3_NPs)—administered at a dose consistent with dietary Mn recommendations (65 mg Mn/kg), on bone tissue processes specifically through the lens of molecular and biochemical regulatory mechanisms. Additionally, the impact of manganese deficiency (complete exclusion of Mn from the mineral mixture added to the diet) on bone metabolism was assessed. In the present study, it was hypothesized that dietary manganese nanoparticles, due to their unique physicochemical properties (such as altered solubility and cellular uptake mechanisms), may modulate bone turnover processes at the molecular level differently than conventional MnCO_3_. Consequently, rather than focusing on biomechanical material properties, this research aimed to elucidate the underlying signaling pathways governing bone remodeling by analyzing the levels of key protein markers and the expression of selected genes related to osteoblast and osteoclast differentiation in the femur and blood of experimental rats. This approach was designed to provide novel mechanistic insights into the role of Mn in bone homeostasis and to evaluate the safety profile of Mn_2_O_3_ nanoparticles at the cellular level. In the present study, only male Wistar rats were used to minimize variability associated with sex-specific hormonal regulation of bone metabolism. In female rodents, the estrous cycle produces rapid fluctuations in estrogen and progesterone concentrations, which markedly influence bone turnover, mineral homeostasis, and bone microarchitecture. These physiological oscillations can obscure treatment-related effects and substantially increase baseline variability, thereby reducing the experimental model’s sensitivity. The use of males, which exhibit a more stable endocrine profile, is therefore a commonly applied approach in mechanistic studies on bone metabolism and mineral supplementation. Future work may extend these observations to female subjects to address potential sex-dependent differences. Additionally, the use of male Wistar rats provides a well-established and reproducible model for studying bone metabolism, as their rapid growth and skeletal remodeling processes are similar to those in humans. The physiological and molecular pathways regulating osteoblast and osteoclast activity in rats are highly homologous to human bone biology, enabling translational understanding of trace element effects. Furthermore, dietary manipulation in rats allows for precise control of manganese intake and nanoparticle exposure, providing a reliable assessment of mechanistic effects. Therefore, the results obtained from this rat model can provide a basis for developing nutritional recommendations for humans and developing nanoparticle-based supplementation strategies.

## 2. Results

### 2.1. Effects of Mn Exclusion from the Mineral Mixture in the Rats’ Diet

**Figure 1:**
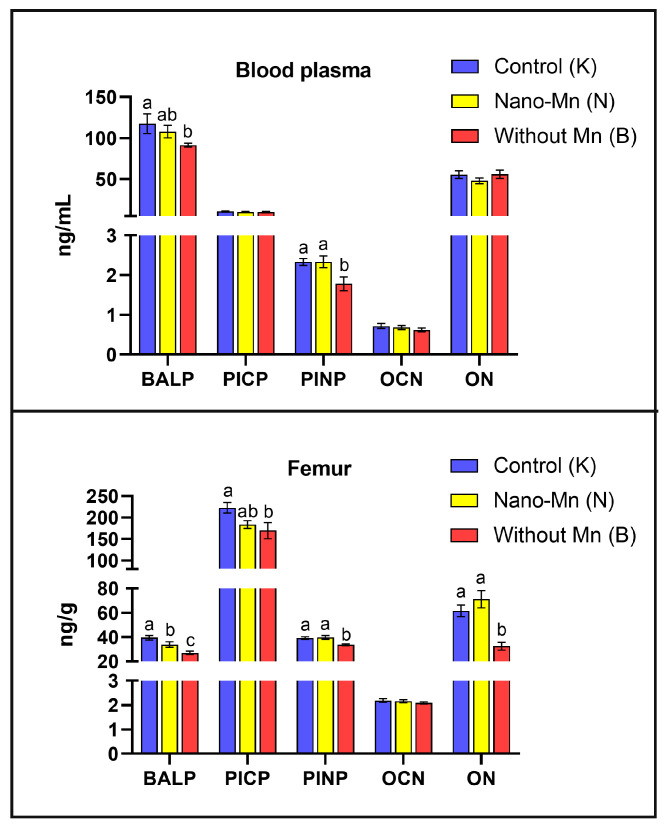
Blood plasma and femur levels of selected bone formation biomarkers (^a–c^—different superscript letters indicate statistically significant differences between groups (*p* < 0.05)). Columns represent the mean values for each experimental group (n = 9) and their variability, expressed as the standard error of the mean (SEM).

**Figure 2:**
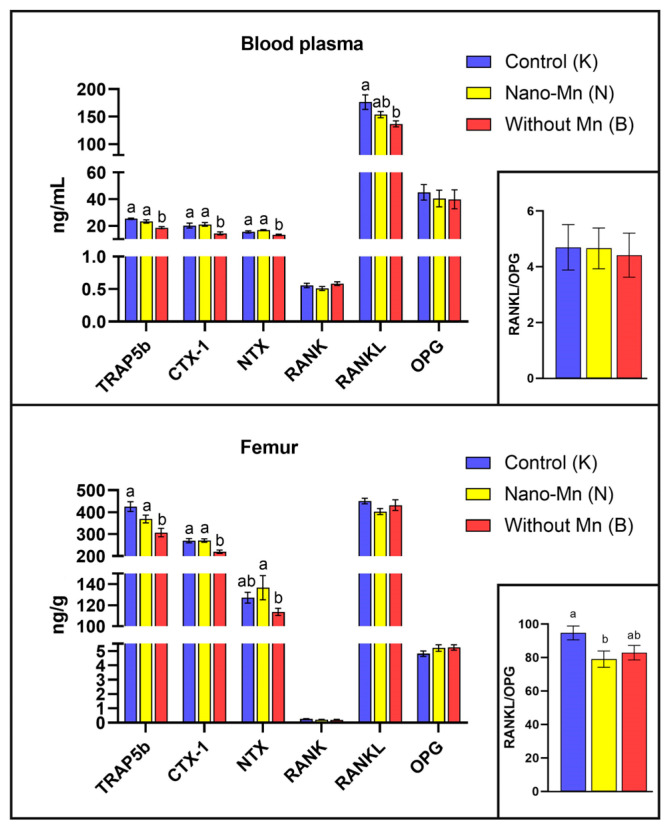
Blood plasma and femur levels of selected bone resorption biomarkers (^a,b^—different superscript letters indicate statistically significant differences between groups (*p* < 0.05)). Columns represent the mean values for each experimental group (n = 9) and their variability, expressed as the standard error of the mean (SEM).

**Figure 3:**
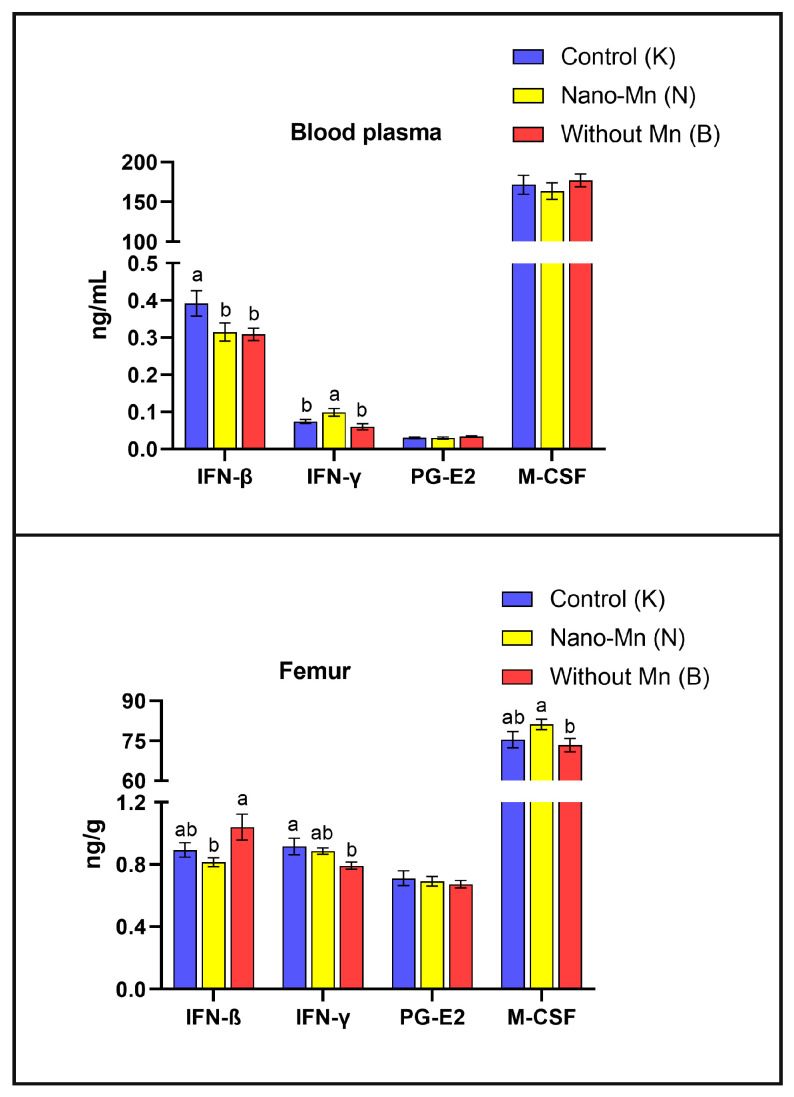
Blood plasma and femur levels of selected inflammatory mediators (^a,b^—different superscript letters indicate statistically significant differences between groups (*p* < 0.05)). Columns represent the mean values for each experimental group (n = 9) and their variability, expressed as the standard error of the mean (SEM).

**Figure 4:**
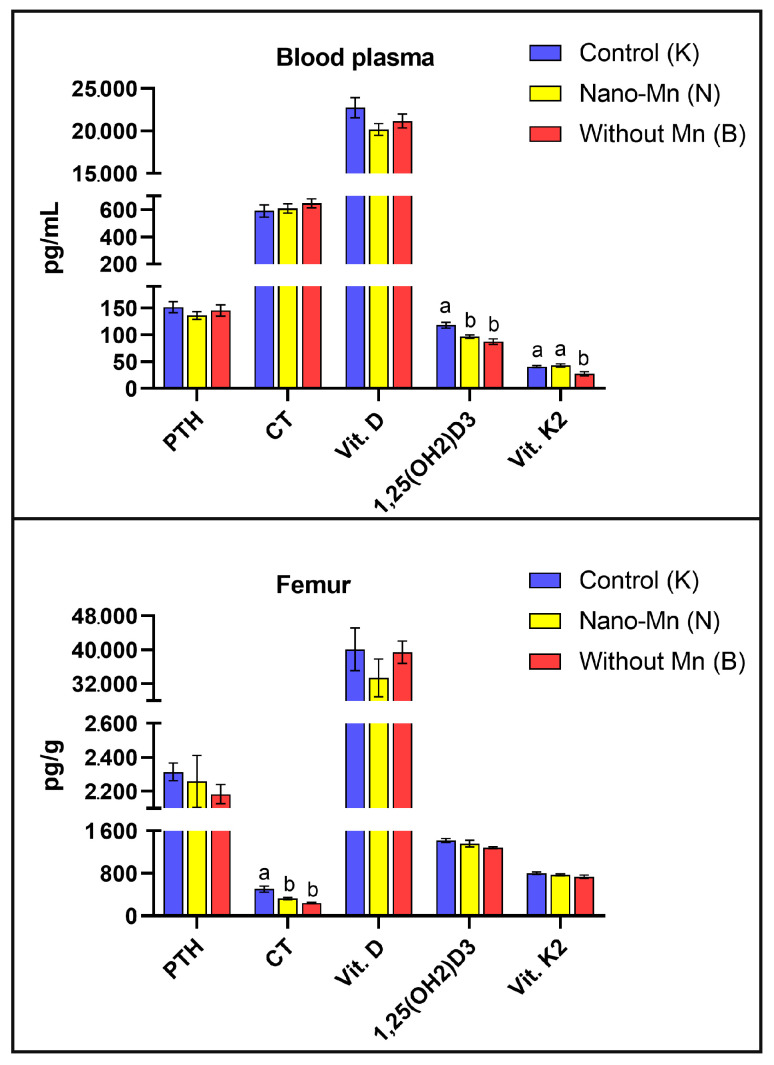
Blood plasma and femur levels of key hormonal and vitamin regulators of bone metabolism (^a,b^—different superscript letters indicate statistically significant differences between groups (*p* < 0.05)). Columns represent the mean values for each experimental group (n = 9) and their variability, expressed as the standard error of the mean (SEM).

The study demonstrated that rats fed a diet completely devoid of manganese in the mineral mixture exhibited significantly lower plasma and femur levels of protein concerning with bone formation such as BALP (*p* = 0.044 and *p* < 0.001, respectively) and PINP (*p* = 0.010 and *p* = 0.001, respectively) compared with control animals receiving the recommended level of Mn in the form of MnCO_3_. In the femur only, osteonectin (ON) level was reduced by 47.38% (*p* < 0.001) and PICP level by 23.84% (*p* = 0.016) relative to controls (Figure 1). Bone-resorption biomarkers followed a similar pattern: both plasma and femur TRAP5b (*p* < 0.001, both) and CTX-1 (*p* = 0.006 and *p* < 0.001, respectively) levels decreased under the influence of manganese deficit in the rats’ diet. Plasma RANKL and NTX levels were also lower in Mn-deficient rats, by 22.45% (*p* = 0.006) and 14.28% (*p* < 0.001), respectively, versus the control group (Figure 2). Furthermore, manganese deficiency altered inflammatory signaling: plasma IFN-β levels were reduced by 21.28% (*p* = 0.043), and femur IFN-γ levels by 13.57% (*p* = 0.026) compared to the control group (Figure 3). Moreover, biomarkers of vitamin regulation of bone metabolism were impaired, with 1,25(OH)_2_D_3_ and vitamin K_2_ plasma levels declining by 26.00% (*p* < 0.001) and 32.73% (*p* = 0.002), respectively, relative to the control group. In the femur of Mn-deficient rats, CT levels were also found to be 51.93% lower (*p* < 0.001) than in those fed the control diet (Figure 4). For clarity, the detailed numerical data supporting these results are presented in the Supplementary Materials (Tables S1–S3).

### 2.2. Effect of Replacing Standard MnCO_3_ with Manganese (III) Oxide Nanoparticles (Mn_2_O_3_NPs) in the Rats’ Diet

**Figure 5:**
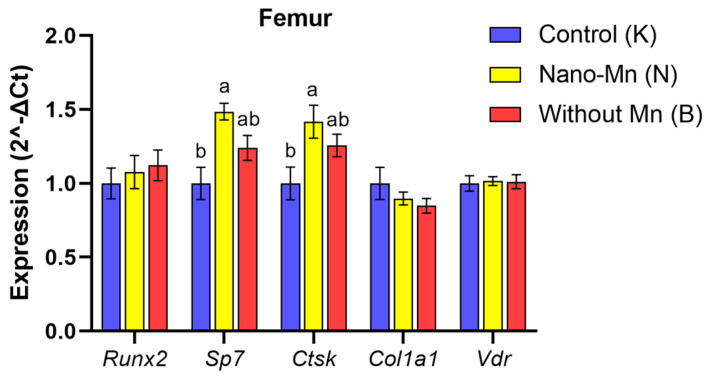
Femur expression profile of key genes involved in osteogenesis and bone remodelling (^a,b^—different superscript letters indicate statistically significant differences between groups (*p* < 0.05)). Columns represent the mean values for each experimental group (n = 9) and their variability, expressed as the standard error of the mean (SEM).

The study demonstrated that inclusion of the recommended manganese level as Mn_2_O_3_NPs in the dietary mineral mixture led to a 14.29% decrease in femur BALP level (*p* < 0.001) compared with control rats receiving the same Mn dose but as standard MnCO_3_ (Figure 1). In the femur of experimental rats, the RANKL/OPG ratio was also reduced by 16.54% (*p* = 0.025) versus the control group (Figure 2). Substituting MnCO_3_ with Mn_2_O_3_NPs also altered the circulating cytokines profile: plasma IFN-γ level increased by 33.45% (*p* = 0.003), while IFN-β level decreased by 19.67% (*p* = 0.043) relative to the control group receiving MnCO_3_ as a dietary Mn source (Figure 3). Additionally, in this group, the plasma 1,25(OH)_2_D_3_ level declined by 17.88% (*p* < 0.001) and the femur CT level decreased by 35.25% (*p* < 0.001) compared to control rats (Figure 4). Mn_2_O_3_NPs supplementation also significantly increased femur gene expression of *Sp7* by 48.5% (*p* < 0.001) and *Ctsk* by 41.8% (*p* = 0.010) compared to the control group (Figure 5). For clarity, the detailed numerical data supporting these results are presented in the Supplementary Materials (Tables S1–S3).

## 3. Discussion

Manganese (Mn) deficiency in the body can lead to several adverse consequences for bone tissue development and function, including impaired bone growth and disrupted bone turnover, which promotes increased bone resorption [2,4]. Strause et al. [5] observed that long-term Mn deficiency led to a decrease in calcium (Ca) concentration in the femurs and an increase in its serum levels in the studied rats, suggesting impaired bone mineralization and enhanced resorption. Moreover, Leach and Muenster [14] demonstrated that Mn deficiency impairs bone tissue synthesis by reducing the level of mucopolysaccharides, particularly those containing galactosamine, both in the epiphyseal cartilage and the organic bone matrix. There are also reports indicating that Mn deficiency during pregnancy may lead to defective skeletal development and growth, joint enlargement, and other skeletal deformities in various animal species, such as rats, mice, pigs, chickens, and sheep [15]. Taskozhina et al. [1] highlight the pivotal role of Mn in regulating osteoblast activity, particularly through its involvement in key signaling pathways such as PI3K/AKT and WNT/β-catenin, as well as in the activation of enzymes crucial for the synthesis of bone matrix components like proteoglycans and collagen. Therefore, Mn deficiency may lead to impaired osteoblast function, delayed osteogenesis, and compromised bone structure. Additionally, a strong link has been established between Mn deficiency and the development of osteoporosis. Patients affected by this condition exhibit significantly reduced plasma manganese levels compared to healthy women, further emphasizing the essential role of manganese in proper collagen synthesis and bone mineralization [2]. Li & Yang [6] emphasize that Mn deficiency in the diet may impair the activity of antioxidant enzymes, particularly manganese-dependent superoxide dismutase (MnSOD). This enzyme converts superoxide radicals into hydrogen peroxide and oxygen, protecting mitochondria from oxidative damage. In the case of Mn deficiency, its synthesis or biological functionality may be impaired, leading to the accumulation of pathological levels of free radicals in metabolically active bone cells, which disrupts the integrity and metabolism of bone tissue [6]. Our findings are consistent with the literature and confirm the negative impact of Mn deficiency on biochemical processes occurring in bone tissue. It was found that limiting Mn intake in the diet of rats significantly reduced the levels of key bone turnover markers such as BALP, TRAP5b, CTX-1, and PINP in both plasma and femur. Additionally, a decrease in NTX and RANKL levels in plasma and PICP in femur was observed. BALP (bone-specific alkaline phosphatase), PINP (procollagen type I N-terminal propeptide), and PICP (procollagen type I C-terminal propeptide) are markers of osteoblast activity [16]. BALP is an isoenzyme of alkaline phosphatase characteristic of the mineralization phase, while PINP and PICP reflect the secretion of type I collagen, a key component of the bone matrix [16]. Their reduced levels indicate decreased osteoblast activity, which may result in impaired collagen synthesis and mineralization disorders, potentially contributing to impaired bone quality. It is worth emphasizing that Mn deficiency also negatively affected other markers associated with osteoblast function. A significant reduction in the plasma concentrations of 1,25(OH)_2_D_3_ and vit. K_2_—compounds crucial for calcium homeostasis and activation of osteocalcin, one of the main proteins secreted by osteoblasts, were observed. Additionally, a decrease in osteonectin levels in the femur was noted—a glycoprotein involved in the mineralization and maturation of the bone matrix [17]. These alterations provide additional evidence of impaired osteoblast function due to Mn deficiency, which, when combined with disrupted collagenogenesis, may contribute to reduced bone quality and structural integrity.

Simultaneously, the conducted study revealed a decrease in the levels of TRAP5b (tartrate-resistant acid phosphatase isoform 5b), NTX (N-terminal telopeptide of type I collagen), and CTX-1 (C-terminal telopeptide of type I collagen)—bone resorption markers associated with osteoclast activity. TRAP5b is an enzyme secreted by osteoclasts during active bone resorption, responsible for breaking down bone matrix proteins and degrading organic phosphates [16]. CTX-1 and NTX, in turn, are fragments of type I collagen produced during its degradation in the course of bone resorption, and their plasma concentrations reflect the intensity of this process [16]. RANKL (Receptor Activator of Nuclear Factor κB Ligand), a cytokine belonging to the TNF family, plays a crucial role in osteoclast differentiation and activation [18]. The observed reduction in the levels of these markers due to Mn deficiency indicates an inhibition of bone resorption, likely caused by impaired osteoclast differentiation or survival. Furthermore, our study demonstrated that Mn deficiency affects immune processes in the body, as evidenced by the decreased levels of IFN-β in plasma and IFN-γ in the bone tissue of the examined rats. Given that these cytokines play a key role in regulating osteoclastogenesis, partly through the STAT1/NFATc1 signaling pathway, their reduced concentrations may further suppress osteoclast activity, contributing to the development of a low-turnover bone phenotype [19].

The concurrent inhibition of both osteoblast and osteoclast activity observed in the present study indicates that manganese deficiency induces a low-turnover bone phenotype rather than merely a unidirectional loss of bone mass. Although the suppression of resorption may initially appear as an adaptive mechanism aimed at preserving bone mass under conditions of impaired formation, in reality, this coupled decline in cellular activity disrupts bone tissue homeostasis. Proper remodeling is essential for microdamage repairing; therefore, its inhibition leads to the accumulation of aged, fragile bone matrix. The conclusion regarding reduced bone turnover under Mn deficiency is strongly supported by the results of our previous studies, which included the measurement of Mn content in the femur and histological analysis of this tissue [20]. Although dietary Mn deficiency in rats did not significantly affect Mn levels in the tissue, it significantly deteriorated its morphological characteristics, as evidenced by intensified fibrous dystrophy and numerous foci of fatty degeneration in the cortical bone, which are directly linked to inhibited osteoblastogenesis [20]. Since mesenchymal stem cells (MSCs) in the bone marrow can differentiate into either osteoblasts or adipocytes [21], it is highly probable that manganese deficiency, by impairing key osteogenic signaling pathways (e.g., Wnt/β-catenin), shifts cell differentiation towards adipogenesis. Consequently, the biochemical profile of markers indicative of reduced bone turnover (lower BALP and TRAP5b levels) is consistent with histopathological findings of fibrous dystrophy and fatty degeneration in the cortical bone [20]. Although these structural changes constitute indirect evidence reflecting secondary consequences of impaired remodeling rather than direct evidence of reduced cellular activity, they nevertheless seem to support the interpretation that manganese deficiency induces a low bone turnover phenotype. However, these morphological changes represent secondary consequences rather than direct evidence of reduced osteoblast and osteoclast activity.. It is also noteworthy that despite considerable changes in the protein levels of bone turnover biomarkers, the expression of genes involved in osteoblast and osteoclast differentiation (*Runx2*, *Sp7*, *Ctsk*, *Col1a1*, *Vdr*) did not exhibit significant alterations in the femur of the examined rats. This suggests that Mn deficiency does not directly affect transcription in bone cells but rather acts at the post-transcriptional or post-translational level, disrupting the modification, secretion, or stabilization of newly synthesized proteins. A potential cause of the observed bone turnover disorders induced by Mn deficiency may be the reduced activity of manganese-dependent superoxide dismutase (MnSOD). Its deficiency impairs the regulation of signaling pathways responsible for the differentiation and function of osteoblasts and osteoclasts [1]. At the same time, MnSOD deficiency increases oxidative stress, which may lead to premature aging and apoptosis of these cells [1,6].

The results of our study confirm that manganese, whose role in bone metabolism is often underestimated compared to calcium or phosphorus, is an essential element for maintaining the balance between osteogenesis and osteoclastogenesis in bone tissue. This highlights the importance of balanced Mn supplementation, particularly in animals with limited dietary diversity and in humans with impaired absorption or metabolism of this micronutrient. However, our findings indicate that dietary supplementation in rats with the recommended level of Mn in the form of manganese(III) oxide nanoparticles (Mn_2_O_3_NPs) led to disturbances in bone metabolism and disrupted the balance of bone remodeling processes—effects that were not observed following supplementation with a conventional inorganic Mn salt (MnCO_3_). Specifically, we observed a significant reduction in BALP levels in the femur, as well as a decrease in 1,25(OH)_2_D_3_ (the active form of vitamin D_3_) in blood plasma. These changes indicate impaired bone formation due to reduced osteoblastic activity. Calcitriol (1,25(OH)_2_D_3_) plays a pivotal role in regulating bone mineralization and enhancing calcium and phosphorus absorption in the small intestine, as thoroughly described in the literature [22,23]. The observed reduction in plasma 1,25(OH)_2_D_3_ levels likely stems from disrupted vitamin D metabolism, as its activation requires sequential hydroxylation: first 25-hydroxylation in the liver by CYP2R1/CYP27A1 enzymes to form 25(OH)D, followed by 1α-hydroxylation in the kidneys by CYP27B1 to yield active 1,25(OH)_2_D_3_ [24,25,26]. A plausible mechanism underlying this impairment is hepatotoxicity and nephrotoxicity induced by Mn_2_O_3_ nanoparticle exposure. Indeed, numerous studies have demonstrated that manganese oxide nanoparticles (MnO_2_-NPs and Mn_2_O_3_-NPs) accumulate primarily in the liver and kidneys, triggering oxidative stress through excessive ROS production, mitochondrial dysfunction, and depletion of antioxidant defenses (GSH, SOD) [27,28,29,30]. Singh et al. [27] reported dose-dependent elevations in serum AST, ALT, and LDH levels in rats exposed to MnO_2_-NPs, accompanied by histopathological evidence of liver necrosis and renal tubular damage. Similarly, Hafez et al. [30] showed that MnO_2_-NPs in mice induced significant increases in ALT, AST, urea, and creatinine levels, along with liver and kidney inflammation. Notably, these effects were partially mitigated by vitamin D supplementation, underscoring the link between Mn-NP toxicity and vitamin D dysregulation [30]. Li et al. [28] demonstrated mitochondrial damage and elevated ROS levels in hepatocytes of broiler chickens exposed to Mn_2_O_3_-NPs, leading to metabolic dysfunction. Given that liver and kidney dysfunction can directly impair the enzymatic conversion of vitamin D precursors, the most probable explanation for the calcitriol deficiency observed in rats receiving dietary Mn_2_O_3_ nanoparticles instead of standard MnCO_3_ is the hepatotoxic and nephrotoxic effects of these nanoparticles.

As a consequence, this may lead to disturbances in calcium-phosphate homeostasis and impaired mineralization of the bone matrix, further supported by the decreased calcitonin levels observed in the experimental rats’ femur. The observed lack of corresponding increases in osteoblast mineralization activity suggests that Mn_2_O_3_NPs may negatively affect the final stages of osteoblast differentiation and maturation, as well as their functional performance, likely through reactive oxygen species (ROS)-dependent pathways or mechanisms related to the nano–bio interface of the administered nanoparticles [13]. Available literature strongly supports the notion that micronutrients administered in the form of nanoparticles, characterized by specific physicochemical parameters (e.g., shape, size, electrochemical potential), exhibit enhanced bioavailability, cellular uptake, and interaction with intracellular signaling pathways compared to their bulk counterparts. This may significantly alter their ultimate biological effects [18,31,32,33,34].

The results of our study indicate, at the molecular level, that dietary supplementation with Mn_2_O_3_ nanoparticles (Mn_2_O_3_NPs) in rats may inhibit not only osteogenic but also osteoclastic processes, as evidenced by a significant reduction in the RANKL:OPG ratio observed in the femur. This observed suppression of the pro-osteoclastic signal likely results from the immunomodulatory effects of Mn_2_O_3_NPs on the immune system. In the plasma of experimental animals, we noted an increased concentration of IFN-γ accompanied by a reduced level of IFN-β, indicating a shift in the immune response towards a Th1 profile. Elevated IFN-γ levels promote a persistent inflammatory state and may interfere with the RANK/RANKL signaling pathway, which is essential for the differentiation and activation of osteoclasts [35]. As a result, osteoclastogenesis may be suppressed, consistent with the observed decrease in the RANKL:OPG ratio. The concurrent reduction in plasma IFN-β, a type I interferon with anti-inflammatory properties, could further diminish the capacity to suppress inflammation and may weaken the bone microenvironment’s antiviral and antiproliferative responses. These changes may impair the function of immunocompetent cells present in the bone marrow and niche, negatively impacting regenerative and remodeling processes in bone tissue [36]. Interestingly, we observed increased expression of both *Sp7* (encoding Osterix—a master transcription factor essential for osteoblast maturation) and *Ctsk* (encoding Cathepsin K—a key osteoclast protease) in the femurs of rats receiving Mn_2_O_3_NPs. These findings likely represent a compensatory transcriptional upregulation attempting to balance bone turnover under conditions of disrupted remodeling, potentially triggered as a feedback response to the suppressed pro-osteoclastic signaling indicated by the reduced RANKL:OPG ratio, which, however, failed to translate into effective bone tissue functionality. Although protein levels were not directly assessed in this study, the discordance between mRNA abundance and active protein levels is well documented in bone biology [37,38]. This discrepancy stems from complex post-transcriptional regulation, including microRNA (miRNA)-mediated silencing, proteasomal degradation via ubiquitination, and ROS-dependent post-translational inhibition [39,40]. Our findings seem to corroborate this dissociation mechanism, as despite overexpression of the *Sp7* and *Ctsk* genes, deterioration in functional markers was observed, specifically, reduced BALP levels and a RANKL:OPG ratio, alongside disturbed bone histological structure [20]. These observations are therefore consistent with the “nano-bio interface” hypothesis, suggesting that Mn_2_O_3_ nanoparticles, by generating oxidative stress, may induce post-translational modifications that impair the maturation and activation of key enzymatic proteins, even in the presence of preserved or enhanced transcription [28]. Furthermore, this functional impairment is likely exacerbated by environmental factors, particularly the elevated IFN-γ level induced by Mn_2_O_3_NPs supplementation. As previously mentioned, IFN-γ, a proinflammatory cytokine, can directly interfere with the RANK/RANKL pathway, thereby limiting osteoclast differentiation and activity. This likely leads to a dissociation between transcriptional stimulation and functional resorptive activity of osteoclasts. Our findings, therefore, suggest that the inclusion of Mn_2_O_3_NPs in the diet may alter bone remodeling dynamics through a dissociation of bone formation and resorption processes, which could have implications for bone quality and integrity and should be verified by direct structural analyses in future studies. This aligns with observations from our previous studies, which showed that Mn_2_O_3_NPs supplementation, despite providing the recommended level of Mn, had no significant effect on femur Mn levels but resulted in deteriorated femur morphology. Supporting this conclusion are histopathological changes, including fibrous dystrophy of the cortical bone and numerous foci of steatosis located both within the marrow cavity and the cortical bone. Those changes were not observed in rats supplemented with the conventional inorganic form of manganese (MnCO_3_) [20].

Given the observed adverse effects of bare Mn_2_O_3_NPs, future translational applications should focus on optimizing nanoparticle formulations to mitigate toxicity while preserving bioavailability. Potential strategies include surface functionalization with biocompatible polymers (e.g., PEGylation, chitosan coating) or protein coronas, which can reduce ROS generation and limit off-target accumulation in the liver and kidneys [41,42,43]. Additionally, controlling size and surface charge (zeta potential) may minimize immune recognition and prevent the proinflammatory Th1 shift observed in this study. Such modifications could enable the safe exploitation of Mn-based nanomaterials for dietary supplementation.

## 4. Materials and Methods

The current study builds upon a broader research initiative investigating the multifaceted biological effects of manganese deficiency and supplementation with manganese(III) oxide nanoparticles. The experimental design, methodological approach, and procedural details have been previously described in scientific articles published by Cholewińska et al. [20], Sołek et al. [44], and Różaniecka-Zwolińska et al. [45].

### 4.1. Characterization of Mn_2_O_3_ Nanoparticles

Manganese(III) oxide nanoparticles (Mn_2_O_3_NPs) were obtained from a commercial supplier (Sky Spring Nanomaterials Inc., Houston, TX, USA). These nanoparticles were chosen based on their high purity (99.9%) and consistent physicochemical properties, including a particle size of 40–60 nm, a density of 7.3 g/cm^3^, a melting point of 1519 K, and a boiling point of 2334 K, as declared by the manufacturer. Before dietary application, the nanoparticles were suspended in a pre-calculated, appropriate amount of rapeseed oil, commonly used as an ingredient in feed mixtures, and then sonicated to ensure proper dispersion and homogeneity. The thus prepared oil-based nanoparticle emulsion was added to the diet after mixing the base formula with the remaining minerals. The hydrodynamic size distribution and zeta potential of the nanoparticles Mn_2_O_3_NPs, determined using dynamic light scattering (DLS) with a Zetasizer Nano ZS (Malvern Instruments, Malvern, UK), was analyzed and published previously [46].

### 4.2. Animal Experiment Design and Feeding Protocol

The experiment was conducted on twenty-seven clinically healthy male albino Wistar rats (Cmdb:WI), aged four weeks at the start of the study. Animals were obtained from the breeding unit of the Institute of Animal Reproduction and Food Research PAS (Olsztyn, Poland; breeder registry no. 051). The rats were randomly assigned to three equal experimental groups (n = 9 per group). Group sizes were determined based on our prior experimental experience to ensure statistically reliable outcomes while adhering to the 3R principles (Replacement, Reduction, Refinement), with each rat serving as an independent experimental unit. Allocation to groups was randomized by generating numbers with MS Excel’s RAND() function (Microsoft Corporation, Redmond, WA, USA, version 2511). The experiment lasted 12 weeks, as this period encompasses the rapid growth phase in rats and allows multiple cycles of bone modeling and remodeling, enabling the detection of diet-induced effects on bone structure and mineralization. To minimize environmental bias, cages were organized so that rats from each treatment group occupied equivalent positions (top, bottom, left, right) on the rack within the housing room. The project manager was the only person with full knowledge of each animal’s group assignment. Analytical contractors were selectively informed, and most remained blinded to treatment allocations. The animals were housed individually in stainless steel cages within a controlled environment (temperature: 21–22 °C; relative humidity: 60 ± 10%; 12-h light/dark cycle; air exchange rate: 15 changes per hour). Standard welfare monitoring was maintained throughout the study. All procedures involving animals were conducted in full compliance with the applicable legal regulations in the Republic of Poland and the European Union Directive 2010/63/EU [47] on the protection of animals used for scientific purposes. The research protocol, including objectives, in vivo study design, and analysis plan, was reviewed and approved by the Local Ethics Committee for Animal Experiments in Olsztyn (Approval No. 13/2022; Olsztyn, Poland; 16 March 2022). The study adhered to the ARRIVE (Animal Research: Reporting of In Vivo Experiments) guidelines [48], and every effort was made throughout the study to minimize discomfort and ensure ethical treatment of all experimental animals. Throughout the 12-week study period, rats were provided with ad libitum access to tap water and semi-purified diets based on a modified AIN-93G-VM casein formulation [49] (details in Table 1). Diets were prepared in advance, stored at 4 °C in hermetically sealed containers, and re-mixed before feeding to maintain homogeneity. Three dietary regimens were tested to evaluate the effects of different treatments with supplemental Mn in the diet: the control group (K; control) received a diet supplemented with 65 mg Mn/kg in the form of manganese carbonate (MnCO_3_), which is an integral component of added directly to the mineral mixture, according to NRC [50]; manganese-deficient group (B; without Mn) received a diet devoid of Mn in the mineral mixture; and nanoparticle-supplemented group (N; Mn-nano): received a diet as the B group but containing 65 mg Mn/kg supplied as manganese(III) oxide nanoparticles (Mn_2_O_3_NPs) dispersed in dietary rapeseed oil). Compositions of the mineral mixtures used in each dietary variant are detailed in Table 2 and Table 3. During the feeding trial, any rat that refused food for more than two days, vocalized in pain for over an hour, showed neurological deficits (such as ataxia or loss of posture), or had blood in its feces for more than 24 h would have been humanely euthanized. Euthanasia, as determined by the institute’s veterinarian, would have been carried out by gradual CO_2_ exposure or, after sedation, cervical dislocation. Based on published data and our own prior work, the risk of these adverse effects from manganese deficit or dietary nanoparticle exposure was negligible, so all rats completed the study.

### 4.3. Biological Sample Acquisition

Throughout the study, no animals were excluded from the experiment. At the end of the 12-week dietary intervention, all animals (n = 9 per group) underwent a 12-h fasting period during which water was provided *ad libitum*. Subsequently, general anesthesia was administered via *intraperitoneal* injection of ketamine (100 mg/kg body weight) and xylazine (10 mg/kg body weight), both dissolved in physiological saline (0.9% NaCl). Upon achieving full anesthesia, a laparotomy was performed, and blood was drawn directly from the caudal vena cava into heparinized tubes (n = 9 per group). Immediately following blood collection, animals were sacrificed by cervical dislocation following approved protocols. Then, the right femur was carefully dissected, freed from surrounding soft tissues, and processed for further experimental procedures (n = 9 per group). Blood samples were centrifuged at 350× *g* for 10 min at 4 °C to separate the plasma, which was subsequently aliquoted and stored at −80 °C for future biochemical analyses. Excised femora were prepared following the methodology described by Nance et al. [51]. Then, they were divided into two parts. One portion was designated for total RNA extraction and rapidly frozen in liquid nitrogen and preserved at −80 °C. The second portion of each dissected femur was used to prepare tissue homogenates for the quantification of bone metabolism-related protein biomarkers, following the protocol described by Cholewińska et al. [18]. The resulting supernatants were carefully transferred to sterile Eppendorf tubes and stored at −80 °C until further analysis.

### 4.4. Measurement of Bone Metabolism-Related Biomarker Levels in Plasma and Femur

The levels of a broad set of biomarkers related to bone metabolism were measured in blood plasma and femur homogenates (n = 9 per group). The determined parameters included bone-specific alkaline phosphatase (BALP), tartrate-resistant acid phosphatase isoform 5b (TRAP-5b), C- and N-terminal propeptides of type I procollagen (PICP and PINP, respectively), C- and N-terminal telopeptides of type I collagen (CTX-I and NTX-I, respectively), osteonectin (ON), osteocalcin (OCN), osteoprotegerin (OPG), RANK and RANKL glycoproteins, prostaglandin E2 (PG-E2), macrophage colony-stimulating factor (M-CSF), interferon-β (IFN-β) and interferon-γ (IFN-γ), parathyroid hormone (PTH), calcitonin (CT), total vitamin D, its biologically active form 1,25-dihydroxyvitamin D_3_ (1,25-OH_2_D_3_), and vitamin K_2_ (vit. K_2_). All determinations were performed using commercial enzyme-linked immunosorbent assay (ELISA) kits according to the manufacturer’s instructions (Shanghai Qayee Biotechnology Co., Ltd., Shanghai, China). Absorbance was recorded at 450 nm using a Sunrise™ microplate reader (Tecan Group Ltd., Männedorf, Switzerland). The RANKL-to-OPG ratio was also computed as an indicator of osteoclastogenic profile.

### 4.5. Assessment of Bone Remodelling-Associated Gene Expression in the Femur

RNA was isolated from previously preserved femur fragments (n = 9 per group) following the procedure described by Cholewińska et al. [18]. The quantity of the isolated RNA was determined spectrophotometrically using a Nabi UV-VIS spectrophotometer (MicroDigital Co., Ltd., Gyeonggi, Republic of Korea), while its integrity was assessed by electrophoresis on a 0.8% agarose gel. For complementary DNA (cDNA) synthesis, 1 µg of RNA was reverse-transcribed using the RevertAid RT Reverse Transcription Kit (Thermo Fisher Scientific, Waltham, MA, USA) according to the manufacturer’s instructions. Primers specific for gene expression analysis—Sp7 transcription factor (*Sp7*), Runx2 transcription factor (*Runx2*), cathepsin K (*Ctsk*), collagen type I alpha 1 chain (*Col1a1*), and vitamin D receptor (*Vdr*)—were designed using Primer3web version 4.1.0 (https://primer3.ut.ee/) and synthesized by Genomed (Warsaw, Poland). These primers were carefully selected to ensure both specificity and amplification efficiency for the target genes. The primer sequences are listed in Table 4. Quantitative real-time PCR was performed on the QuantStudio 7 Pro Real-Time PCR System (Thermo Fisher Scientific, Waltham, MA, USA) using SYBR™ Select Master Mix (Thermo Fisher Scientific, Waltham, MA, USA). The thermal cycling protocol consisted of an initial UNG activation step at 50 °C for 2 min, followed by a 10-min initial denaturation at 95 °C. This was followed by 40 amplification cycles, each including denaturation at 95 °C for 30 s, annealing at 48–56 °C for 30 s (temperature varied depending on the gene), and elongation at 72 °C for 45 s. Negative controls without cDNA template were included to verify the absence of contamination. Melting curve analysis was conducted from 50 °C to 72 °C with intervals of 0.3 °C per second to assess product specificity. All PCR reactions were performed in duplicate. Gene expression levels were normalized and calculated using the 2^^-ΔCt^ method, with glyceraldehyde-3-phosphate dehydrogenase (*Gapdh*), β-actin (*Actb*), and 18S ribosomal RNA (*18S rRNA*) serving as endogenous reference genes.

### 4.6. Statistical Analysis

All results are expressed as the mean with the standard error of the mean (SEM). Statistical differences among the experimental groups—Control (K), Nano-Mn (N), and Without Mn (B)—were evaluated using one-way analysis of variance (ANOVA), followed by appropriate post hoc multiple comparison tests, conducted with the Statistica software version 14.1.0 (TIBCO Software Inc., Tulsa, OK, USA).

## 5. Study Limitations

The present study was specifically designed as a mechanistic investigation into the molecular and biochemical pathways altered by manganese deficiency and nanoparticle exposure. As such, it has several inherent limitations that must be acknowledged. First, the exclusive use of a single rodent strain and sex may limit the generalizability of our findings to different genetic backgrounds and female physiology. Second, and most importantly, while we assessed key molecular markers and gene expression, as well as referenced qualitative histology from our previous studies [20], direct functional and structural assessments of bone material properties, specifically micro-computed tomography, dynamic histomorphometry with fluorescent labeling, biomechanical testing, and immunohistochemical validation of protein targets, were not performed in this specific dataset. Consequently, our conclusions regarding “bone quality” are inferred from biochemical data and should be interpreted as hypothesis-generating evidence of molecular dysregulation rather than definitive proof of mechanical failure. Finally, extrapolation of these rat data to human bone biology should be undertaken with caution. Although rats are a well-established model for human bone biology, species-specific differences in manganese metabolism should be noted. Rats absorb and excrete manganese more efficiently than humans, and their faster metabolic rate can influence tissue distribution and bone accumulation. Despite these differences, the fundamental role of Mn in bone formation is conserved, supporting the translational relevance of the model while cautioning against direct extrapolation of absolute doses.

## 6. Conclusions

To conclude, the results of our study indicate that dietary manganese deficiency is associated with decreased markers of both bone formation and resorption, which may consequently disrupt the dynamic balance of bone remodeling. This was accompanied by changes in mineral metabolism and biomarkers related to bone matrix mineralization, potentially compromising bone structure and strength. Furthermore, in the setting of this study, replacing standard manganese (MnCO_3_) supplementation with Mn_2_O_3_ nanoparticles (Mn_2_O_3_NPs) did not result in the expected improvement in bone turnover and instead was associated with a deterioration in markers of osteogenic and resorptive processes. Because these conclusions are based on indirect indicators of bone metabolism, such as biochemical markers and gene expression, they should be interpreted cautiously and confirmed in future studies using direct structural and functional analyses of bone tissue, including assessment of micro-computed tomography, dynamic histomorphometry, biomechanical testing, and immunohistochemical study.

## Figures and Tables

**Table 1:** Composition of basal experimental diet fed to rats, % (This table is also included in the articles published by Cholewińska et al. [20], Sołek et al. [44] and Różaniecka-Zwolińska et al. [45]).

Ingredient	Content
Unchangeable ingredients:	
Casein ^1^	14.8
DL-methionine	0.2
Cellulose ^2^	8.0
Choline chloride	0.2
Rapeseed oil	8.0
Cholesterol	0.3
Vitamin mix ^3^	1.0
Maize starch ^4^	64.0
Changeable ingredient:	
Mineral mix (MX) ^5^	3.5
Calculated content:	
Crude protein	13.5

^1^ Casein preparation: crude protein 89.7%, crude fat 0.3%, ash 2.0%, and water 8.0%. ^2^ α-Cellulose (SIGMA, Poznan, Poland), the main source of dietary fibre. ^3^ AIN-93G-VM [49], g/kg mix: 3.0 nicotinic acid, 1.6 Ca pantothenate, 0.7 pyridoxine-HCl, 0.6 thiamin-HCl, 0.6 riboflavin, 0.2 folic acid, 0.02 biotin, 2.5 vitamin B-12 (cyanocobalamin, 0.1% in mannitol), 15.0 vitamin E (all-rac-α-tocopheryl acetate, 500 IU/g), 0.8 vitamin A (all-trans-retinyl palmitate, 500,000 IU/g), 0.25 vitamin D-3 (cholecalciferol, 400,000 IU/g), 0.075 vitamin K-1 (phylloquinone), 974.655 powdered sucrose. ^4^ Maize starch preparation: crude protein 0.6%, crude fat 0.9%, ash 0.2%, total dietary fibre 0%, and water 8.8%. ^5^ Changeable dietary ingredient to manganese level; mineral mixture (the base according to NRC [50]) with standard Mn level and deprived of Mn, see Table 2 and Table 3.

**Table 2:** Experimental schema * (provided manganese dosage was calculated taking into account MnCO_3_ in MX or Mn from Mn_2_O_3_ nanoparticles (Mn_2_O_3_NPs) preparation) (This table is also included in the articles published by Cholewińska et al. [20], Sołek et al. [44] and Różaniecka-Zwolińska et al. [45]).

Group	12 Weeks of Feeding
**B** (Negative CONT, without Mn in MX)	A diet with MX deprived of Mn (n = 9)
**K** (Control, with standard supplementation of Mn in MX)	A diet containing 65 mg/kg Mn from MnCO_3_ (n = 9)
**N** (Nano Mn, with standard supplementation of Mn but from a novel nanoparticle source)	A diet containing 65 mg/kg Mn from Mn_2_O_3_ nanoparticles (n = 9)

n = 9, number of rats used in a particular feeding period. * Experimental groups: B—during all twelve weeks of feeding the Mn deficient rats were given a diet with MX deprived of Mn (MnCO_3_ excluded from MX); K—the rats were fed a diet with standard mineral mixture (MX) resulting in 65 mg Mn (from MnCO_3_ in MX) per 1 kg of a diet during 12 weeks of feeding; N—the rats were given a diet containing 65 mg/kg Mn from Mn_2_O_3_ nanoparticles preparation per 1 kg of a diet during 12 weeks of feeding.

**Table 3:** Composition of mineral mixtures (MX) used in experimental diets (This table is also included in the articles published by Cholewińska et al. [20], Sołek et al. [44] and Różaniecka-Zwolińska et al. [45]).

	MX with Standard Mn Dosage ^1^	MX Deprived of Mn ^2^
Calcium carbonate anhydrous CaCO_3_	357	357
Potassium phosphate monobasic K_2_HPO_4_	196	196
Potassium citrate C_6_H_5_K_3_O_7_	70.78	70.78
Sodium chloride NaCl	74	74
Potassium sulphate K_2_SO_4_	46.6	46.6
Magnesium oxide MgO	24	24
Microelements mixture	18	18
Starch	To 1000 g = 213.62	To 1000 g = 213.62
Microelements mixture:		
Ferric citrate [16.7% Fe]	31	31
Zinc carbonate ZnCO_3_ [56% Zn]	4.5	4.5
Manganous carbonate MnCO_3_ [44.4% Mn]	23.4	0
Copper carbonate CuCO_3_ [55.5% Cu]	1.85	1.85
Potassium iodate KJ	0.04	0.04
Citric acid C_6_H_8_O_7_	To 100 g = 39.21 g	To 100 g = 62.61

^1^ given to the K group (12 weeks of feeding), ^2^ given to the B and N groups (12 weeks of feeding), but the N group was provided with the appropriate amount of Mn from Mn_2_O_3_ nanoparticles preparation as an emulsion along with dietary rapeseed oil.

**Table 4:** Primer sequences for targeted genes.

Gene	Primer	Sequence (5′-3′)	Melting Temperature (°C)	Product Size (nt)	Gen Bank Access No.
*Gapdh*	Forward	AACGGGAAGCTCACTGGCATG	63.3	305	NM_017008.4
	Reverse	TCCACCACCCTGTTGCTGTAG	62.0		
*Actb*	Forward	GAAGATCAAGATCATTGCTCCT	55.9	111	NM_031144.3
	Reverse	TACTCCTGCTTGCTGATCCACA	61.4		
*18sRNA*	Forward	ACTCAACACGGGAAACCTCA	59.2	114	NR_046237.3
	Reverse	AATCGCTCCACCAACTAAGA	56.6		
*Sp7*	Forward	CTGGGAAAAGGAGGCACAAAGA	60.8	166	NM_001037632.1
	Reverse	GGGGAAAGGGTGGGTAGTCATT	61.7		
*Runx2*	Forward	GTGCGGTGCAAACTTTCTCC	60.3	102	NM_001278483.2
	Reverse	AATGACTCGGTTGGTCTCGG	59.8		
*Ctsk*	Forward	TCTCACATTCCTTCCTCAACAG	57.7	150	NM_031560.2
	Reverse	GACTCCAGCGTCTATCAGCAC	60.5		
*Col1a1*	Forward	CAGTCGATTCACCTACAGCACG	61.3	201	NM_053304.1
	Reverse	GGGATGGAGGGAGTTTACACG	60.1		
*Vdr*	Forward	ACAGTCTGAGGCCCAAGCTA	60.5	103	NM_017058.2
	Reverse	TCCCTGAAGTCAGCGTAGGT	60.3		

*Gapdh*, glyceraldehyde-3-phosphate dehydrogenase; *Actb*, β-actin; *18S rRNA*, 18S ribosomal RNA; *Sp7*, Sp7 transcription factor (Osterix); *Runx2*, Runx2 transcription factor; *Ctsk*, cathepsin K; *Col1a1*, collagen type I alpha 1 chain; *Vdr*, vitamin D receptor.

## Data Availability

All data generated or analyzed during this study are included in this published article and the Supplementary Materials.

## Supplementary Materials

The following supporting information can be downloaded at: https://www.mdpi.com/article/10.3390/ijms27010153/s1.

## Abbreviations

The following abbreviations are used in this manuscript:

Mn_2_O_3_NPs	manganese (III) oxide nanoparticles
BALP	bone-specific alkaline phosphatase
TRAP-5b	tartrate-resistant acid phosphatase isoform 5b
PICP	C-terminal propeptides of type I procollagen
PINP	N-terminal propeptides of type I procollagen
CTX-I	C-terminal telopeptides of type I collagen
NTX	N-terminal telopeptides of type I collagen
ON	osteonectin
OCN	osteocalcin
OPG	osteoprotegerin
RANK	RANK glycoprotein
RANKL	RANKL glycoprotein
RANKL:OPG	RANKL:OPG rato
PG-E2	Prostaglandin E2
M-CSF	macrophage colony-stimulating factor
IFN-β	interferon-β
IFN-γ	interferon-γ
PTH	parathyroid hormone
CT	calcitonin
vit. D	total vitamin D
1,25-OH_2_D_3_	1,25-dihydroxyvitamin D_3_ (calcitriol)
vit. K_2_	vitamin K_2_
