# Supplementary material for: Manganese Deficiency and Mn2O3 Nanoparticles Supplementation Disrupt Bone Remodeling and Mineral Matrix Maturation in Rats
Source: Int J Mol Sci. 2025 Dec 23;27(1):153. doi: 10.3390/ijms27010153 (PMC12786009; doi:10.3390/ijms27010153)
Supplement: Supplementary file 1 [file ijms-27-00153-s001.zip › Supplementary materials Table S1.pdf]

Table S1. Bone metabolism-related parameters in the blood plasma.

	Control (K)	Nano-Mn (N)	Without Mn (B)	SEM	P-value
BALP, ng/mL	117.5[a]	107.9[ab]	91.44[b]	5.048	0.044
TRAP5b, ng/mL	25.27[a]	23.24[a]	18.56[b]	0.745	<0.001
PICP, ng/mL	10.63	9.901	9.759	0.415	0.437
PINP, ng/mL	2.334[a]	2.334[a]	1.782[b]	0.093	0.010
CTX-1, ng/mL	20.15[a]	21.04[a]	14.32[b]	1.023	0.006
NTX, ng/mL	15.41[a]	16.71[a]	13.21[b]	0.443	<0.001
ON, ng/mL	55.53	48.01	55.97	2.554	0.240
OCN, pg/mL	718.0	682.8	618.1	31.417	0.234
OPG, ng/mL	45.16	40.43	39.88	3.583	0.589
RANK, pg/mL	557.4	509.6	582.1	18.369	0.134
RANKL, ng/mL	176.4[a]	153.7[ab]	136.8[b]	5.850	0.006
RANKL:OPG	4.696	4.662	4.417	0.433	0.814
PG-E2, pg/mL	30.36	29.66	33.63	1.373	0.279
M-CSF, ng/mL	171.5	163.4	177.1	5.787	0.384
IFN-β, pg/mL	392.0[a]	314.9[b]	308.6[b]	16.400	0.043
IFN-γ, pg/mL	74.06[b]	98.83[a]	59.94[b]	5.534	0.003
PTH, pg/mL	151.8	136.0	145.4	5.372	0.274
CT, pg/mL	589.7	608.3	646.0	21.455	0.331
Vit. D, ng/mL	22.75	20.19	21.17	0.552	0.074
1,25$(OH_2)D_3$, pg/mL	117.8[a]	96.74[b]	87.22[b]	3.545	<0.001
Vit. K_2, pg/mL	40.85[a]	43.01[a]	27.48[b]	2.167	0.002

SEM, pooled standard error of mean (standard deviation for all rats divided by the square root of rat number, n=27);

[a,b] Mean values within a row with unlike superscript letters are shown to be significantly different (P<0.05);

BALP, bone-specific alkaline phosphatase; TRAP-5b, tartrate-resistant acid phosphatase isoform 5b; PICP, C-terminal propeptides of type I procollagen; PINP, N-terminal propeptides of type I procollagen; CTX-I, C-terminal telopeptides of type I collagen; NTX, N-terminal telopeptides of type I collagen; ON, osteonectin; OCN, osteocalcin; OPG, osteoprotegerin; RANK, RANK glycoprotein; RANKL, RANKL glycoprotein; RANKL:OPG, RANKL:OPG rato; PG-E2, prostaglandin E2; M-CSF, macrophage colony-stimulating factor; IFN-β, interferon-β; IFN-γ, interferon-γ; PTH, parathyroid hormone; CT, calcitonin; vit. D, total vitamin D, 1,25-OH_2D_3, 1,25-dihydroxyvitamin D_3 (calcitriol); vit. K_2, vitamin K_2.
