# Supplementary material for: Manganese Deficiency and Mn2O3 Nanoparticles Supplementation Disrupt Bone Remodeling and Mineral Matrix Maturation in Rats
Source: Int J Mol Sci. 2025 Dec 23;27(1):153. doi: 10.3390/ijms27010153 (PMC12786009; doi:10.3390/ijms27010153)
Supplement: Supplementary file 1 [file ijms-27-00153-s001.zip › Supplementary materials Table S2.pdf]

Table S2. Bone metabolism-related parameters in the femur.

	Control (K)	Nano-Mn (N)	Without Mn (B)	SEM	P-value
BALP, ng/g	39.46^{a}	33.82^{b}	27.00^{c}	1.452	<0.001
TRAP5b, ng/g	425.8^{a}	369.2^{a}	307.5^{b}	14.532	<0.001
PICP, ng/g	222.7^{a}	183.7^{ab}	169.6^{b}	8.901	0.016
PINP, ng/g	39.21^{a}	39.74^{a}	33.63^{b}	0.856	0.001
CTX-1, ng/g	270.4^{a}	271.3^{a}	219.6^{b}	6.729	<0.001
NTX, ng/g	127.1^{ab}	136.6^{a}	113.6^{b}	4.561	0.049
ON, ng/g	61.55^{a}	71.07^{a}	32.39^{b}	4.352	<0.001
OCN, pg/g	2186.4	2154.6	2087.4	34.649	0.287
OPG, ng/g	4.809	5.198	5.235	0.119	0.174
RANK, pg/g	260.5	214.0	202.0	19.259	0.255
RANKL, ng/g	450.5	402.9	432.0	10.500	0.082
RANKL:OPG	94.66^{a}	79.00^{b}	82.87^{ab}	2.787	0.025
PG-E2, pg/g	711.4	691.2	673.2	19.961	0.480
M-CSF, ng/g	75.42^{ab}	81.17^{a}	73.37^{b}	1.544	0.048
IFN-β, pg/g	894.2^{ab}	814.2^{b}	1040.1^{a}	36.720	0.013
IFN-γ, pg/g	916.2^{a}	886.0^{ab}	791.9^{b}	22.260	0.026
PTH, pg/g	2314.5	2258.2	2183.7	55.961	0.387
CT, pg/g	504.7^{a}	326.8^{b}	242.6^{b}	29.623	<0.001
Vit. D, ng/g	40.10	33.41	39.46	2.385	0.294
1,25$(OH_2)D_3$, pg/g	1418.4	1358.4	1284.6	26.574	0.051
Vit. K_2, pg/g	803.1	775.4	735.9	15.296	0.093

SEM, pooled standard error of mean (standard deviation for all rats divided by the square root of rat number, n=27); ^{a,b} Mean values within a row with unlike superscript letters are shown to be significantly different (P<0.05);

BALP, bone-specific alkaline phosphatase; TRAP-5b, tartrate-resistant acid phosphatase isoform 5b; PICP, C-terminal propeptides of type I procollagen; PINP, N-terminal propeptides of type I procollagen; CTX-I, C-terminal telopeptides of type I collagen; NTX, N-terminal telopeptides of type I collagen; ON, osteonectin; OCN, osteocalcin; OPG, osteoprotegerin; RANK, RANK glycoprotein; RANKL, RANKL glycoprotein; RANKL:OPG, RANKL:OPG rato; PG-E2, prostaglandin E2; M-CSF, macrophage colony-stimulating factor; IFN-β, interferon-β; IFN-γ, interferon-γ; PTH, parathyroid hormone; CT, calcitonin; vit. D, total vitamin D, 1,25-OH_2D_3, 1,25-dihydroxyvitamin D_3 (calcitriol); vit. K_2, vitamin K_2.
