# Supplementary material for: Manganese Deficiency and Mn2O3 Nanoparticles Supplementation Disrupt Bone Remodeling and Mineral Matrix Maturation in Rats
Source: Int J Mol Sci. 2025 Dec 23;27(1):153. doi: 10.3390/ijms27010153 (PMC12786009; doi:10.3390/ijms27010153)
Supplement: Supplementary file 1 [file ijms-27-00153-s001.zip › Supplementary materials Table S3.pdf]

Table S3. Expression of bone remodeling-related genes in the femur.

	Control (K)	Nano-Mn (N)	Without Mn (B)	SEM	P-value
Sp7	1.000[b]	1.485[a]	1.240[ab]	0.062	<0.001
Runx2	1.000	1.077	1.123	0.060	0.451
Ctsk	1.000[b]	1.418[a]	1.258[ab]	0.065	0.010
Col1a1	1.000	0.898	0.849	0.043	0.183
Vdr	1.000	1.016	1.011	0.025	0.815

SEM, pooled standard error of mean (standard deviation for all rats divided by the square root of rat number, n=27);

[a,b] Mean values within a row with unlike superscript letters are shown to be significantly different (P<0.05);

Sp7, Sp7 transcription factor (Osterix); *Runx2*, Runx2 transcription factor; *Ctsk*, cathepsin K; *Col1a1*, collagen type I alpha 1 chain; *Vdr*, vitamin D receptor
